# Paramyxoviruses in Bats in Poland—The First Detection

**DOI:** 10.3390/pathogens15020223

**Published:** 2026-02-17

**Authors:** Anna Orłowska, Karol Stasiak, Jerzy Rola, Marcin Smreczak

**Affiliations:** Department of Virology and Viral Animal Diseases, National Veterinary Research Institute, Al. Patyzantów 57, 24-100 Puławy, Poland; karol.stasiak@piwet.pulawy.pl (K.S.); jrola@piwet.pulawy.pl (J.R.)

**Keywords:** paramyxoviruses, bats, prevalence, Poland, phylogenetics, haplotypes

## Abstract

Bats are hosts to many diseases that emerge in humans and livestock. Knowledge about the diversity and circulation of paramyxoviruses in European bat populations, despite their recognized importance, remains limited. Here, we present data on the first detection of paramyxoviruses in Poland in the new bat species of *Cnephaeus serotinus* and *Cnephaeus nilsonii*, which have never been previously recognized as paramyxovirus hosts, as well as in *Myotis daubentonii* and two unknown bat species. Viral RNA was detected in fecal and intestinal samples using the semi-nested RT-PCR protocol followed by Sanger sequencing. A widespread comprehensive phylogenetic study supported by haplotype network analyses of 376 nt sequences of paramyxoviruses detected in bats worldwide revealed that paramyxoviruses are closely related to the host and strongly correlate to the area of origin.

## 1. Introduction

Bats constitute the second most abundant mammalian order after rodents, with over 1400 recognized species worldwide [1,2]. They comprise approximately 22% of all named animal species [3], with extensive species diversity, common roosting, and wide distribution strictly connected with mobility behavior. Their exceptional species diversity, wide geographical distribution, longevity, and gregarious behavior make them important hosts of numerous viral pathogens, as demonstrated by large-scale studies worldwide. They are hosts for highly dangerous zoonotic viruses such as lyssaviruses, Hendra virus, Nipah virus, Ebola virus, and SARS-CoV-1 and SARS-CoV-2 coronaviruses, which can be transmitted to humans and animals [4,5,6,7,8]. They account for most novel viral sharing events and are likely to share viruses that promote emerging infectious diseases in humans [9]. The number of infectious viruses increases proportionately to the total number of viruses maintained by each reservoir group, which, in turn, is explained by the number of animal species within each group. Variation in the number of zoonoses among animal groups therefore arises as a consequence of their species richness; thus, the preponderance of rodent- or bat-associated zoonoses could reflect the large number of rodent and bat species relative to other mammalian groups [10]. Most human pathogens, including Marburg virus, Nipah virus, Hendra virus, Ebola virus, SARS-CoV, and Middle East respiratory coronavirus (MERS-CoV), most probably originated in bats [5,6,7,8]. Although coronavirus diversity seems to be higher in bats than in any other mammal, and despite the fact that SARS-CoV, MERS-CoV, and HCoV-229E exist in bats, identical human pathogens such as human SARS have never been isolated in bats, despite intensive attempts [11]. Direct infection from bats to humans seems to be rare according to a serological survey of 128 people with prolonged and close contact with Pteroid bats, and in whom no evidence of infection with Hendra virus was detected. Bats can harbor viruses closely related to the human pathogens that might have been the ancestral origin of human viruses; however, it is likely that these viruses need an intermediate host in which the viral mutations occur and where the virus will reach significant prevalence for zoonotic spillover to humans [11].

*Paramyxoviridae* is a large family of viruses comprising four subfamilies and fourteen genera based on genetic distances, with an additional three viruses not assigned to a genus or subfamily [12]. It includes some of the most important human and animal viruses, such as measles, distemper, mumps, parainfluenza, Newcastle disease, respiratory syncytial virus, and metapneumoviruses. Diseases caused by paramyxoviruses in humans lead to high mortality and morbidity across the world [13] despite the availability of pre- and/or post-exposure treatment. Henipaviruses belonging to the *Orthoparamyxovirinae* subfamily within *Paramyxoviridae* are responsible for life-threatening diseases in humans, horses, and pigs, but Morbilliviruses are causative agents of canine distemper and ruminant Rinderpest [14].

Paramyxoviruses have been identified in a variety of hosts including birds (chickens and turkeys), aquatic animals (salmon, whale, seal, dolphin, and porpoise), rodents (mice and rats), dogs, cats, sheep, reptiles (snake and lizards), horses, cattle, pigs, simians, humans, and bats [15,16,17]. They are common in fruit bat populations in Africa, Australia, South America, Asia, and Madagascar. Infections with paramyxoviruses in bat populations in Europe have so far been found in Germany, Luxembourg, Bulgaria, and Italy, with a general low prevalence [14,18,19,20,21,22]. In Europe, paramyxoviruses were found in Myotis bats including *M. daubentonii*, *M. mystacinus*, *M. myotis*, *M. nattereri*, *M. alcathoe*, *M. emarginatus*, and *M. bechsteinii*, all of which occur in Poland.

Paramyxoviruses are able to transmit across species due to the antigenic and immunopathologic similarity of human respiratory syncytial virus (HRSV) and bovine respiratory syncytial virus (BRSV) [23]. Similarly, human parainfluenza virus 3 and 1 (HPIV-3 and HPIV-1) and bovine parainfluenza virus 3 (BPIV-3) are genetically related, while in guinea pigs, human parainfluenza virus 3 with 95.6–97.9% nucleotide identity was detected in colony of guinea pigs in Japan in 1998 [24,25]. The transmission occurs via respiratory droplets or direct contact, and paramyxoviruses switch hosts at a rate higher than other RNA viruses such as those belonging to the *Alphavirus*, *Flavivirus*, and *Rhabdoviridae* genera [26]. The synanthropic habitats of many bat species and the relatively high level of host switching of paramyxoviruses increases the risk of transmission from bats to other potential hosts, including humans.

Most paramyxovirus species have been identified in large Old World fruit bats (*Yinpterochiroptera* suborder) and Eurasian bats, whereas few studies have been performed in the *Yangochiroptera* suborder [27,28,29,30]. Despite their recognized importance, knowledge about the diversity and circulation of paramyxoviruses in European bat populations remains limited. There is a lack of data on the occurrence of paramyxoviruses associated with bats in central–western Europe. Surveillance studies focusing on bat populations are essential for improving the understanding of paramyxovirus ecology and diversity. Studying the diversity of bat viruses is particularly significant due to bats’ evolutionary history. Therefore, the main aim of the study was to investigate the prevalence of paramyxoviruses in bats in Poland and their diversity in order to extend the overall knowledge on viral circulation in bats.

## 2. Materials and Methods

### 2.1. Samples

A total of 274 bat samples (80 oral swabs, 110 fecal samples, and 84 intestinal samples) were collected between 2012 and 2022 from the country of Poland. Samples were collected from bats of both sexes and included individuals belonging to ninety species and thirty-four unidentified bats (Table 1). Oral swabs and sixty bat fecal samples were collected from bats captured for faunistic surveys and population monitoring by qualified chiropterologists. Oral swabs were collected by swabbing the oral cavity of the bats with the Copan Universal transport (UTM-RT) System, using a flexible applicator swab with flocked nylon fiber (Copan, Brescia, Italy). Samples of feces were caught in cotton bags and transferred to 5 mL Eppendorf tubes. All sample tubes were marked with a unique identification number immediately after sampling and stored under chilled conditions using frozen transport cooling packs until delivery to the laboratory. The sampling methods were harmonized through multiple sampling occasions. Bat trapping and biometric measurements were performed with permission from the General Directorate of Environmental Protection in Warsaw. Bat species and sex were determined morphologically on the basis of estimation of characteristics and genitalia. The remaining fifty fecal samples consisted of guano samples collected from bat roosting sites of *Rhinolophus hipposideros* located at Silesia voivodeship (one of 16 regions of Poland), where feces were collected from aluminum foil placed on the floor of the bat colony. The foil was placed in the evening, and the guano was collected in individual tubes the next morning. The intestinal samples were collected from the necropsy of found-dead bats (n = 84) obtained across Poland and sent to the National Veterinary Research Institute for rabies surveillance. For found-dead bats, the molecular identification of bat species was performed according to the protocol described in Harris et al. [31]. In the laboratory, oral swabs, feces, and intestinal samples were kept frozen at −80 °C until RNA extraction.

### 2.2. Processing of Sample and RNA Extraction

Viral RNA was extracted from individual samples using the QIAamp Viral RNA Mini Kit (Qiagen, Hilden, Germany) following the manufacturer’s protocol, and eluted in a final volume of 60 µL. All extraction runs included controls: the RNA of bovine parainfluenza (BPIV) field virus, confirmed by sequencing, as a positive control and transport medium as a negative control.

### 2.3. RT-PCR Assays and Sequencing

Viral RNA was used as a template in a semi-nested RT-PCR assay that amplified a product within the RNA polymerase (L) gene, as described by Tong et al. (2008) [15]. Each reverse transcription reaction and the first round of PCR amplification was performed using the SuperScript III One-Step RT-PCR System with Platinum Taq DNA Polymerase (Invitrogen, Carlsbad, CA, USA) in a total volume of 25 µL. The mixture consisted of 1× reaction mix buffer (Invitrogen, Carlsbad, CA, USA), 400 nM of each primer (forward: 5′-GAA GGI TAT TGT CAI AAR NTN TGG AC-3′; reverse: 5′-GCY GAA GTT ACI GGI TCI CCD ATR TTN C-3′), 200 µM of deoxynucleotide triphosphate mix, 1 µL MgCl_2_, 0.1 µL RNase inhibitor, 1 µL of SuperScript™ III RT/Platinum™ Taq Mix (Invitrogen, Carlsbad, CA, USA), and 5 µL of template RNA. In the first round of amplification, reverse transcription was performed in a Biometra Thermocycler (Biometra, Göttingen, Germany) using the following cycling conditions: 60 °C for 1 min for denaturation, cDNA synthesis at 50 °C for 30 min, and pre-denaturation at 95 °C for 15 min, followed by 40 cycles of denaturation at 95 °C for 15 s, primer annealing at 50 °C for 30 s, elongation at 72 °C for 1 min, and a final elongation step at 72 °C for 10 min. The second round of PCR reactions was performed in the same thermocycler using the Invitrogen Platinum Taq DNA Polymerase kit (Thermo Fisher Scientific, Carlsbad, CA, USA). The reaction mixture (25 µL) contained 2 µL PCR product, 400 nM of the forward primer (5′-GTT GCT TCA ATG GTT CAR GGN GAY AA-3′), the same reverse primer used in the first round, 0.2 mM deoxynucleotide mix (Sigma-Aldrich, St. Louis, MO, USA), and 1 µL MgCl_2_ in 1× buffer. The mixtures were initially denatured at 94 °C for 2 min, followed by 40 cycles of 94 °C for 30 s, 50 °C for 30 s, 72 °C for 1 min, and a final elongation at 72 °C for 10 min. Amplified products were subjected to electrophoresis through a 1.5% GelRed-stained agarose gel for 30 min at 90 V. Negative non-template controls were included with each run.

All PCR reactions containing products of the expected sizes were purified (ExoSAP-IT PCR Product Cleanup Reagent, Thermo Fisher Scientific, Santa Clara, CA, USA) and sequenced in both directions with the same primers used for amplification using BigDye^®^ Terminator version 3.1 (Applied Biosystems, Vilnius, Lithuania) on a 3730 xl DNA Analyzer at Genomed (Warsaw, Poland).

### 2.4. Sequence Analyses

The forward and reverse sequences obtained from each amplicon were trimmed to an identical length (414 bp) and assembled using BioEdit software v.7.2.5. Only sequences that generated high-quality data were included for analysis, while those poorly aligned were removed. The consensus sequences were aligned with the reference sequence of Bat-PV-17770 (GenBank accession number NC_076625.1) using the CLUSTAL W algorithm from the Molecular Evolutionary Genetics Analysis software package, version 10 (MEGAX) [32]. A phylogenetic tree was constructed using the maximum likelihood method with 1000 bootstrap replicates using the General Time Reversible model in MEGAX. The sequence identity (%) among sequences analyzed in the study was calculated using the identity matrix in BioEdit and illustrated as heatmaps using the Sequence Demarcation Tool Version 1.3 (SDTv1.3).

### 2.5. Haplotype Network

All nucleotide sequences were used as an input file to generate a haplotype list in the DnaSP software (version 6.12.03). The haplotypes representative of bat paramyxoviruses were exported in nexus format with trait blocks added to represent geographical location (country) and host. The minimum spanning haplotype networks were produced using default parameters in PopART version 1.7 (available from http://popart.maths.otago.ac.nz, accessed on 4 December 2025). An analysis of molecular variance (AMOVA) was carried out in PopART using the “Simple AMOVA” command to test for correlation between the population genetic structure of obtained sequences and selected traits. The strength of correlation was shown by a PhiST value, with 0 indicating no correlation and 1 indicating perfect correlation. The corresponding *p* values were generated by reference to 1000 random permutations of the input data.

### 2.6. GenBank Accession Numbers

The nucleotide sequences of paramyxoviruses identified in bats in Poland during this study were submitted to GenBank under the following accession numbers: PX734724–PX734727.

## 3. Results

### 3.1. Infection with Bat Paramyxoviruses

**Figure 1:**
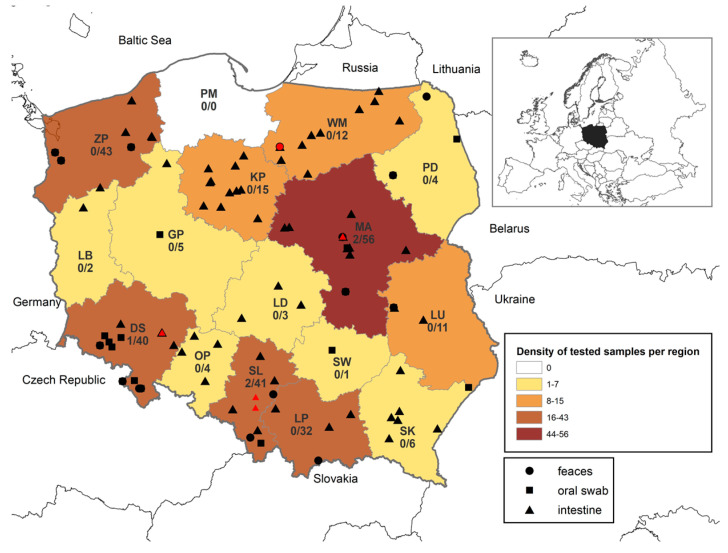
Distribution of bat samples included in the study. Different kinds of samples (feces, oral swab, and intestine) per voivodeship and RT-PCR results (black: negative, red: positive) are shown. The number of positive vs. total samples per region are indicated in each voivodeship. Abbreviations: MA (Masovia), LS (Lower Silesia), WP (Greater Poland), SL (Silesia), PM (Pomerania), LD (Łódź), MP (Lesser Poland), ZP (West Pomerania), LB (Lubusz), KP (Kuyavian–Pomeranian), OP (Opole), PD (Podlaskie), SW (Świętokrzyskie), WM (Warmian–Masurian), PK (Subcarpathian), LU (Lublin).

Extensive research on a group of 274 samples from bats collected from different geographical regions of Poland (Figure 1), including oral swabs (n = 80), fecal samples (n = 110), and bat intestines (n = 84), revealed the presence of bat paramyxoviruses RNA in 5 out of 274 (1.8%) tested samples. Sanger sequencing and further phylogenetic analysis of RT-PCR amplicons confirmed the presence of specific RNA in four samples. Bat paramyxoviruses were found in 3 out of 15 voivodeships (regions of Poland): Mazovia (n = 2), Silesia (n = 2), and Lower Silesia (n = 1), located in southern and central Poland (Figure 1). Viral RNA was found in the intestine samples of Serotine bats: *C. serotinus* (n = 1; 3.6% of tested *C. serotinus*), *C. nilssonii* (n = 1; 50% of tested *C. nilssonii*), feces of *Myotis daubentonii* (n = 1; 3.1% of tested *M. daubentonii*), and in two intestine samples of individuals of bat species that could not be determined through morphological or molecular identification (5.9% of tested unknown bats). The composition of the tested bat species and the prevalence of paramyxoviruses in individual bat species are presented in Table 1.

### 3.2. Molecular and Phylogenetic Analyses

**Figure 2:**
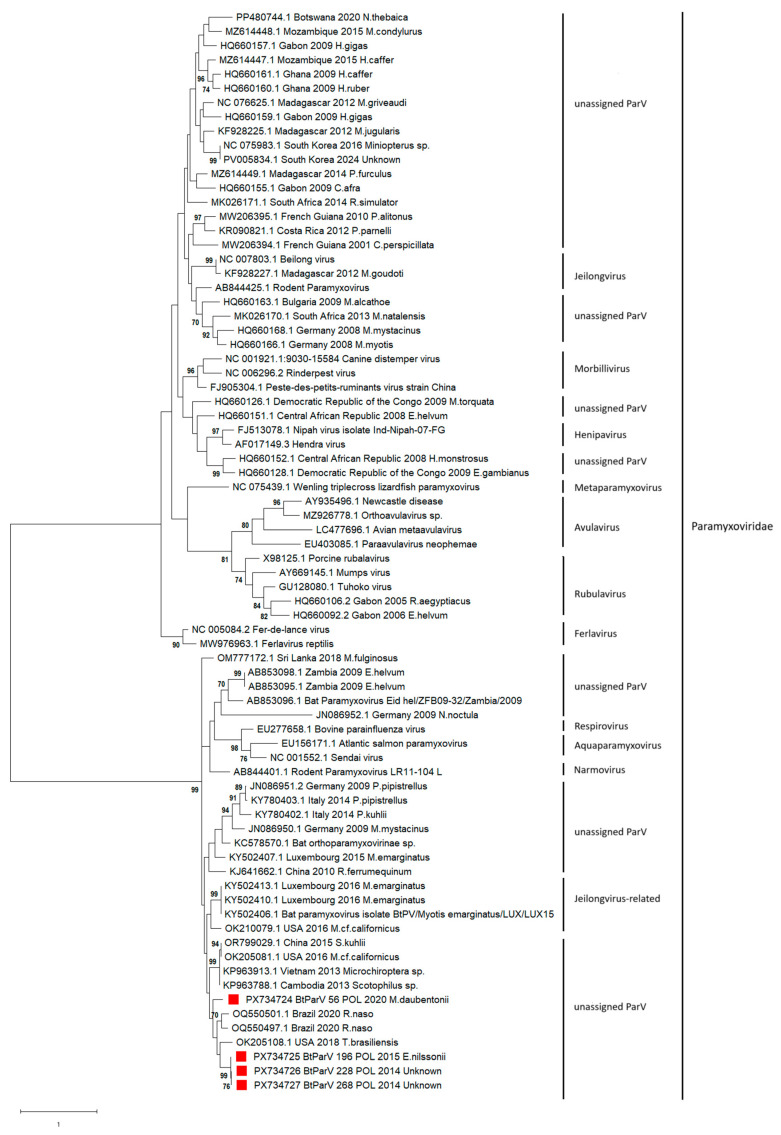
Phylogenetic tree of representatives of paramyxoviruses derived from bats worldwide and paramyxoviruses detected in Poland based on a 369 bp fragment of the RNA polymerase (L) gene. The Polish bat paramyxovirus sequences obtained in the study are labeled BtParV_sample_ID_POL (red square) and _year_of_isolation. Accession numbers for sequences from GenBank are listed for each sequence. The evolutionary history was inferred using the maximum likelihood method and the Tamura–Nei model. The tree with the highest log likelihood (−12,049.79) is shown. The percentage of trees in which the associated taxa clustered together is shown next to the branches. Initial trees for the heuristic search were obtained automatically by applying Neighbor-Join and BioNJ algorithms to a matrix of pairwise distances estimated using the Tamura–Nei model, and then selecting the topology with superior log likelihood value. A discrete Gamma distribution was used to model evolutionary rate differences among sites (5 categories (+G, parameter = 1.6272)). The tree is drawn to scale, with branch lengths measured in the number of substitutions per site. This analysis involved 52 nucleotide sequences. All positions with less than 90% site coverage were eliminated; i.e., fewer than 10% alignment gaps, missing data, and ambiguous bases were allowed at any position (partial deletion option). There were a total of 360 positions in the final dataset. Evolutionary analyses were conducted in MEGA X. Bootstraps over 70% are indicated in the tree branches, supporting the reliability of the tree topology.

The sequence alignments of the partial RNA polymerase (L) region from four Polish bat paramyxoviruses showed 76.5% to 99.7% identity at the nucleotide level. The degree of identity between bat paramyxoviruses detected in Poland (BtParVs) and all reference nucleotide sequences of bat paramyxoviruses isolated worldwide and included in the phylogenetic analysis ranged between 23.6 and 99.7%. Paramyxoviruses isolated in Poland belonged to a group of unassigned paramyxoviruses originating from Europe (Luxembourg, Italy, and Germany), Asia (Sri Lanka, China, Vietnam, and Cambodia), Africa (Zambia), North America (USA), and South America (Brazil), as well as Jeilongvirus-related paramyxoviruses detected in Luxembourg in *M. emarginatus* in 2015 and 2016 (Figure 2) with a nucleotide identity within the group of between 46.8 and 99.7%. Polish isolates were the most similar to paramyxoviruses isolated in Brazil in 2020 (78.1 to 80.4%), the USA in 2018 (75.0–77.0%), and China in 2010 (73.4–74.2%) whereas the nt identity between paramyxoviruses isolated in Poland and those detected in Luxembourg in 2016 from *M. emarginatus* ranged from 71.8 to 76.5%. A slightly lower nucleotide sequence identity was found with paramyxoviruses detected in 2014 in Italy in bats of the species *P. kuhlii* (66.9–70.7%), as well as in Germany in 2009 (67.8–72.2%) in *P. pipistrellus*, *M. mystacinus*, and *N. noctula*.

Significantly lower nt identity was observed for paramyxoviruses isolated in Myotis bats (*M. daubentonii*, *M. mystacinus*, *M. myotis*, *M. nattereri*, and *M. bechsteinii*) in Germany in 2008 and 2014, ranging from 31.3 to 40%, and for paramyxoviruses isolated in Bulgaria in 2009 in *M. alcathoe* (38.4 to 48.7%). Paramyxoviruses isolated in Germany in Myotis bats in 2008 and 2014, as well as those detected in Bulgaria, belonged to large group (over 300 nt sequences) of paramyxoviruses isolated mainly in Asia (South Korea, China), Africa (Madagascar, Mozambique, Ghana, Gabon, Botswana, Democratic Republic of the Congo, and Central African Republic), Central America (Costa Rica), and South America (French Guiana).

The phylogenetic relationships between the paramyxoviruses detected in Poland and representatives of paramyxoviruses identified worldwide are presented in Figure 2. A phylogenetic tree of 376 bat paramyxoviruses available in the GenBank database based on the partial (369 bp) RNA polymerase (L) region and a heatmap showing the percentage of identity are presented in Figures S1 and S2. A separate phylogenetic tree generated for a fragment of the L gene of European paramyxoviruses is shown in Figure S3.

### 3.3. Haplotype Network Analysis

The four Polish and 372 international bat paramyxoviruses’ partial RNA polymerase (L)-region nt sequences (402 bp) formed 297 haplotypes (Figure S4). None of the haplotypes contained sequences from both Poland and international sources. The minimum spanning haplotype networks of the genetic structure amongst 370 sequences showed a visible correlation between the bat paramyxoviral nt sequences and the hosts from which they were isolated (PhiST = 0.73, *p* = <0.001), as well as the country of origin (PhiST = 0.70, *p* = <0.001) (Table 2).

## 4. Discussion

Previously performed studies on bat populations in Poland revealed that they are the hosts of several zoonotic viruses such as lyssaviruses, hantaviruses, coronaviruses including severe acute respiratory syndrome coronavirus (SARS-like CoV), and astroviruses, for which zoonotic potential has been reported [33,34,35,36,37]. Additionally, studies performed with a particular emphasis on Europe demonstrated the presence of paramyxoviruses in a wide range of bat species, including those habituating in Poland, such as Myotis and Pipistrelle bats as well *N. noctula* [20,21,22]. Due to the close proximity of bat habitats to humans, particularly those of synanthropic bat species, they may serve as a host and potential ancestral viral source of paramyxoviruses for humans, especially species that are known to forage in and around human settlements.

Paramyxoviruses primarily spread via respiratory droplets but have also been detected in feces, anal swabs, and urine samples collected from bats and other animal species [9,20,22]. Studies performed on free-ranging European bats revealed the presence of paramyxoviruses in pooled organ samples including lung and kidney tissues of individual bats. Moreover, histopathological examination revealed interstitial nephritis and pneumonia with edematous fluid in the lung parenchyma, moderate follicular hyperplasia of the spleen, and marked leucocytostasis in most blood vessels [21]. Previous studies analyzing paramyxoviruses have also demonstrated that kidney samples are more frequently positive for paramyxoviruses than other bat organs [16,38,39], a fact that has been confirmed by the nephrotropic nature of Hendra and Nipah virus infections [40,41] and orthoparamyxovirus from the genus *Macrojêvirus* detected in neotropical bats [30]. In our study, paramyxoviruses were found in intestines and fecal samples with an overall prevalence of 1.8%, which is comparable to findings of other European bat studies ranging from 1.1% in Luxembourg to 3.9% in northwest Italy [20,22]. The detection of paramyxoviruses in fecal and intestinal samples only may suggest that the intestines are another potential preferred organ in bats for the replication and detection of paramyxoviruses, alongside the kidneys, and feces may constitute an additional route of paramyxoviruses shedding, apart from urine and the respiratory route.

Bats are considered major hosts of mammalian paramyxoviruses with over 60 potential viral species worldwide [16]. The majority of paramyxoviruses have been identified in fruit bats, whereas considerably fewer studies have been conducted in insectivorous bat species. The testing of bat samples collected from Costa Rica, Panama, Brazil, Gabon, Congo, Democratic Republic of the Congo, Central African Republic, Ghana, Germany, Bulgaria, and Romania has identified paramyxoviruses in all countries covered by the study. Among European bat species, paramyxoviruses were detected in Myotis bats (*M. bechsteinii*, *M. daubentonii*, *M. myotis*, *M. mystacinus*, *M. alcathoe*, and *M. capaccini*), with the majority being detected in *M. mystacinus* (five positives/55 tested) [16]. Novel paramyxoviruses were identified in an *M. emarginatus* colony in Luxembourg (Jeilongvirus-related PV), as well as in *M. mystacinus*, *P. pipistrellus*, and *N. noctula* bats in Germany (unclassified paramyxoviruses) and *P. kuhlii* in Italy (unclassified paramyxoviruses) [21]. In the present study, we identified paramyxoviruses in the Serotine bat *C. serotinus* and in *C. nilsonii*, with the latter being a bat species habituating in Europe that has not previously been reported as a host of paramyxoviruses. We also confirmed the circulation of paramyxoviruses in *M. daubentonii*; however, for two bat samples that tested positive for paramyxoviruses, neither morphological nor molecular species identification could be performed. An examination of paramyxoviruses detected in European bats revealed a high degree of genetic diversity and phylogenetic clustering in two distinct groups (Supplementary Figure S3). The lowest nucleotide identity of paramyxoviruses identified in Poland was for viruses originating from Germany that belonged to huge group of paramyxoviruses (Figure S1), whereas paramyxoviruses detected in Poland, Luxembourg, northwestern Italy and two paramyxoviruses identified in Germany in 2009 in *N. noctula* and *M. mystacinus* clustered within smaller common group of paramyxoviruses predominantly isolated in Europe. The fact that most paramyxoviruses detected to date in Europe, often in the same host species, cluster within a common phylogenetic group suggests the presence of a host–virus relationship among paramyxoviruses and a strict correlation between paramyxoviral lineages and their geographic origin. This hypothesis is supported by the results of the haplotype network analysis performed for 376 paramyxovirus nt sequences, including all nucleotide sequences currently available in GenBank, which indicates a strong correlation between the nt sequences of paramyxoviruses and the countries in which they were detected, as well as the correlation between paramyxoviruses and the host. The high diversity of European paramyxoviruses is largely attributable to the nature of their RNA genome, which is highly variable due to the low fidelity of the viral RNA polymerase and the lack of proofreading and repair mechanisms during replication. The increase in genetic variability of paramyxoviruses in bats is certainly affected by the bats’ behaviors, such as common hibernation and gathering in breeding colonies, which facilitates virus transmission and co-infection within colonies and among individuals.

In conclusion, despite growing interest in bats as hosts of viral zoonotic pathogens, particularly emerging viruses, knowledge regarding the presence and diversity of paramyxoviruses in bat populations remains limited. Here, we presented the first report of the shedding of paramyxoviruses by *C. serotinus*, *C. nilsonii*, *M. daubentonii*, and two unidentified bat species in Poland, suggesting that *C. serotinus* and *C. nilsonii* represent previously unrecognized hosts of paramyxoviruses. Widespread comprehensive phylogenetic analyses supported by haplotype network analyses of 376 nt sequences of paramyxoviruses detected in bats worldwide suggest that paramyxoviruses are closely related to the host and strongly correlate to the area of origin.

## Figures and Tables

**Table 1:** The prevalence of paramyxoviruses in different bat species included in the study.

Species	No. of Samples	No. of Positives	Positives % (95% CI)
*Cnephaeus serotinus*	28	1	3.6% (0.2–17.7)
*Cnephaeus nilssonii*	2	1	50.0% (2.6–97.4)
*Nyctalus noctula*	27	0	0% (0.0–12.5)
*Barbastella barbastellus*	5	0	0% (0.0–43.4)
*Plecotus auritus*	14	0	0% (0.0–21.5)
*Vespertillo murinus*	8	0	0% (0.0–32.4)
*Pipistrellus pygmaeus*	16	0	0% (0–19.4)
*Pipistrellus pipistrellus*	7	0	0% (0–35.4)
*Pipistrellus* spp.	5	0	0% (0.0–43.4)
*Pipistrellus nathusii*	8	0	0% (0.0–32.4)
*Myotis dasycneme*	5	0	0% (0.0–43.2)
*Myotis daubentonii*	31	1	3.1% (0.2–15.7)
*Myotis mystacinus*	5	0	0% (0–43.4)
*Myotis nattereri*	10	0	0% (0–27.8)
*Myotis bechsteinii*	2	0	0% (0–82.2)
*Myotis alcathoe*	1	0	0% (0–94.9)
*Myotis brandti*	4	0	0% (0–49.0)
*Myotis emarginatus*	1	0	0% (0–94.9)
*Rhinolophus hipposideros*	61	0	0% (0–5.9)
n/a	34	2	5.9% (1.0–19.1)
Total	274	5	1.8% (0.8–4.2)

n/a—not identified.

**Table 2:** Analysis of molecular variance (AMOVA) results indicating the strength of correlation (PhiST) between the population genetic structure of bat paramyxoviruses and selected traits based on partial RNA polymerase (L) sequence.

Test	Variation Within Populations	Variation Among Populations	Fixation Index (PhiST)	*p* Value
Host ^1^	26.5%	73.5%	0.73	<0.001
Country ^2^	29.7%	70.3%	0.70	<0.001

^1^ *C*. *breviacuda*; *C*. *perspicillata*; *C*. *leucogaster*; *C*. *afra*; *C*. *kibomalandy*; *D*. *rotundus*; *E*. *helvum*; *E*. *gambianus*; *E*. *nilssonii*; *G*. *soricina*; *H*. *caffer*; *H*. *gigas*; *H*. *pomona*; *H*. *ruber*; *Hipposideros* sp.; *H*. *phayrei*; *H*. *monstrosus*; *Microchiroptera* sp.; *M*. *cf ambohitrensis*; *M*. *fulginosus*; *M*. *gleni*; *M*. *griveaudi*; *M*. *mahafaliensis*; *M*. *natalensis*; *M*. *sororculus*; *Miniopterus* sp.; *M*. *condylurus*; *M*. *leucostigma*; *M*. *midas*; *M*. *francoismoutoui*; *M*. *jugularis*; *M*. *torquata*; *M*. *alcathoe*; *M*. *bechsteinii*; *M*. *bombinus*; *M*. *californicus*; *M*. *daubentonii*; *M*. *emarginatus*; *M*. *goudoti*; *M*. *horsfieldii*; *M*. *myotis*; *M*. *mystacinus*; *M*. *nattereri*; *N*. *nanus*; *N*. *thebaica*; *N*. *laticaudatus*; *O*. *madagascariensis*; *P*. *furculus*; *P*. *alitonsus*; *P*. *parnellii*; *P*. *rufus*; *R*. *ferrumequinum*; *R*. *simulator*; *Rhinolophus* sp.; *R*. *naso*; *R*. *aegyptiacus*; *S*. *kuhlii*; *Scotophilus* sp.; *T*. *brasiliensis*; *T*. *menamena*; Unknown. ^2^ Botswana; Brazil; Bulgaria; Cambodia; Central African Republic; China; Comoros; Costa Rica; Democratic Republic of the Congo; Gabon; Germany; Ghana; French Guiana; Japan; Laos; Luxembourg; Madagascar; Mozambique; Poland; Republic of Congo; Reunion; South Africa; South Korea; Sri Lanka; USA; Vietnam; Zambia.

## Data Availability

The nucleotide sequences of Polish Bat-PV described in this study were submitted to GenBank under the following accession numbers: PX734724-PX734727.

## Supplementary Materials

The following supporting information can be downloaded at https://www.mdpi.com/article/10.3390/pathogens15020223/s1, Figure S1. Phylogenetic tree of bat paramyxoviruses (Bat-PVs) based on 369 bp fragment from RNA polymerase (L) gene. Figure S2. Identity matrix for bat paramyxoviruses (Bat-PVs) used in the study. Heatmap showing percent identity between pairs of Bat-PV partial RNA polymerase (L) sequences. Figure S3. Phylogenetic tree presenting all paramyxoviruses isolated in Europe including ParVs detected in Poland based on 369 bp fragment from RNA polymerase (L) gene. Figure S4. International haplotype network analysis based on 370 sequences of partial (402 bp) RNA polymerase (L) of bat paramyxoviruses (Bat-PVs).

## References

[1] Ciechanowski M. (2020). Rząd: Nietoperze—Chiroptera. Zoologia, Ssaki.

[2] Taylor M. (2019). Bats: An Illustrated Guide to All Species.

[3] Letko M., Seifert S.N., Olival K.J., Plowright R.K., Munster V.J. (2020). Bat-borne virus diversity, spillover and emergence. Nat. Rev. Microbiol..

[4] Wang L.F., Eaton B.T. (2007). Bats, civets and the emergence of SARS. Curr. Top. Microbiol. Immunol..

[5] Decaro N., Lorusso A. (2020). Novel human coronavirus (SARS-CoV-2): A lesson from animal coronaviruses. Vet. Microbiol..

[6] Kalpin K., Rota P. (2014). A Review of Hendra Virus and Nipah Virus Infections in Man and Other Animals. Zoonoses—Infections Affecting Humans and Animals.

[7] Shipley R., Wright E., Selden D., Wu G., Aegerter J., Fooks A.R., Banyard A.C. (2019). Bats and Viruses: Emergence of Novel Lyssaviruses and Association of Bats with Viral Zoonoses in the EU. Trop. Med. Infect. Dis..

[8] Leroy E.M., Epelboin A., Mondonge V., Pourrut X., Gonzalez J.P., Muyembe-Tamfum J.J., Formenty P. (2009). Human Ebola outbreak resulting from direct exposure to fruit bats in Luebo, Democratic Republic of Congo 2007. Vector Borne Zoonotic Dis..

[9] Su H., Wang Y., Han Y., Jin Q., Yang F., Wu Z. (2023). Discovery and characterisation of novel paramyxoviruses from bat samples. Virol. Sin..

[10] Mollentzea N., Streicker D.G. (2020). Viral zoonotic risk is homogenous among taxonomic orders of mammalian and avian reservoir hosts. Proc. Natl. Acad. Sci. USA.

[11] Weinberg M., Yovel Y. (2022). Revising the paradigm: Are bats really pathogen reservoirs or do they posses an efficient immune system?. iScience.

[12] ICTV. https://ictv.global/report/chapter/paramyxoviridae/paramyxoviridae.

[13] Park G.Y.S., Tishkowski K. (2023). Paramyxovirus. StatPearls.

[14] Beltz L.A. (2018). Bats and Human Health Ebola, SARS, Rabies and Beyond.

[15] Tong S., Chern S.W., Li Y., Pallansch M.A., Anderson L.J. (2008). Sensitive and broadly reactive reverse transcription-PCR assays to detect novel paramyxoviruses. J. Clin. Microbiol..

[16] Drexler J.F., Corman V.M., Müller M.A., Maganga G.D., Vallo P., Binger T., Gloza-Rausch F., Cottontail V.M., Rasche A., Yordanov S. (2012). Bats host major mammalian paramyxoviruses. Nat. Commun..

[17] Mortlock M., Geldenhuys M., Keith M., Rademan R., Swanepoel L.H., Von Maltitz E.F., Kearney T., Markotter W. (2025). Paramyxo- and coronavirus diversity and host associations in non-volant small mammals: Evidence of viral sharing. Virus Evol..

[18] Suu-Ire R., Obodai E., Bel-Nono S.O., Ampofo W.K., Mazet J.A.K., Goldstein T., Johnson C.K., Smith B., Boaatema L., Asigbee T.W. (2022). Surveillance for potentially zoonotic viruses in rodent and bat populations and behavioral risk in an agricultural settlement in Ghana. One Health Outlook.

[19] Wilkinson D.A., Temmam S., Lebarbenchon C., Lagadec E., Chotte J., Guillebaud J., Ramasindrazana B., Héraud J.M., de Lamballerie X., Goodman S.M. (2012). Identification of novel paramyxoviruses in insectivorous bats of the Southwest Indian Ocean. Virus Res..

[20] Pauly M., Pir J.B., Loesch C., Sausy A., Snoeck C.J., Hübschen J.M., Muller C.P. (2017). Novel Alphacoronaviruses and Paramyxoviruses Cocirculate with Type 1 and Severe Acute Respiratory System (SARS)-Related Betacoronaviruses in Synanthropic Bats of Luxembourg. Appl. Environ. Microbiol..

[21] Kurth A., Kohl C., Brinkmann A., Ebinger A., Harper J.A., Wang L.F., Mühldorfer K., Wibbelt G. (2012). Novel paramyxoviruses in free-ranging European bats. PLoS ONE.

[22] Rizzo F., Edenborough K.M., Toffoli R., Culasso P., Zoppi S., Dondo A., Robetto S., Rosati S., Lander A., Kurth A. (2017). Coronavirus and paramyxovirus in bats from Northwest Italy. BMC Vet. Res..

[23] Baker J.C. (1991). Human and bovine respiratory syncytial virus: Immunopathologic mechanisms. Vet. Q..

[24] Ellis J.A. (2010). Bovine parainfluenza-3 virus. Vet. Clin. N. Am. Food Anim. Pract..

[25] Brabb T., Newsome D., Burich A., Hanes M. (2012). The Laboratory Rabbit, Guinea Pig, Hamster, and Other Rodents.

[26] Kitchen A., Shackelton L.A., Holmes E.C. (2010). Family level phylogenies reveal modes of macroevolution in RNA viruses. Proc. Natl. Acad. Sci. USA.

[27] Sasaki M., Setiyono A., Handharyani E., Rahmadani I., Taha S., Adiani S., Subangkit M., Sawa H., Nakamura I., Kimura T. (2012). Molecular detection of a novel paramyxovirus in fruit bats from Indonesia. Virol. J..

[28] Thibault P.A., Watkinson R.E., Moreira-Soto A., Drexler J.F., Lee B. (2017). Zoonotic potential of emerging Paramyxoviruses: Knowns and unknowns. Adv. Virus Res..

[29] Amman B.R., Albariño C.G., Bird B.H., Nyakarahuka L., Sealy T.K., Balinandi S., Schuh A.J., Campbell S.M., Ströher U., Jones M.E.B. (2015). A recently discovered pathogenic Paramyxovirus, Sosuga virus, is present in *Rousettus aegyptiacus* fruit bats at multiple locations in Uganda. J. Wildl. Dis..

[30] de Souza W.M., Fumagalli M.J., Carrera J.P., de Araujo J., Cardoso J.F., de Carvalho C., Durigon E.L., Queiroz L.H., Faria N.R., Murcia P.R. (2021). Paramyxoviruses from neotropical bats suggest a novel genus and nephrotropism. Infect. Genet. Evol..

[31] Harris S.L., Johnson N., Brookes S.M., Hutson A.M., Fooks A.R., Jones G. (2008). The application of genetic markers for EBLV surveillance in European bat species. Dev. Biol..

[32] Tamura K., Dudley J., Nei M., Kumar S. (2007). MEGA4: Molecular Evolutionary Genetics Analysis (MEGA) software version 4.0. Mol. Biol. Evol..

[33] Orłowska A., Smreczak M., Potyrało P., Trębas P., Bomba A., Rola J. (2021). First Detection of Bat Astroviruses (BtAstVs) Among Bats in Poland: The Genetic BtAstVs Diversity Reveals Multiple Co-Infection of Bats with Different Strains. Viruses.

[34] Smreczak M., Orłowska A., Marzec A., Trębas P., Müller T., Freuling C.M., Żmudziński J.F. (2018). Bokeloh bat lyssavirus isolation in a Natterer’s bat, Poland. Zoonoses Public Health.

[35] Orłowska A., Smreczak M., Freuling C.M., Müller T., Trębas P., Rola J. (2020). Serological Survey of Lyssaviruses in Polish Bats in the Frame of Passive Rabies Surveillance Using an Enzyme-Linked Immunosorbent Assay. Viruses.

[36] Orłowska A., Smreczak M., Thor K., Niedbalska M., Pawelec D., Trębas P., Rola J. (2022). The Genetic Characterisation of the First Detected Bat Coronaviruses in Poland Revealed SARS-Related Types and Alphacoronaviruses. Viruses.

[37] Dafalla M., Orłowska A., Keleş S.J., Straková P., Schlottau K., Jeske K., Hoffmann B., Wibbelt G., Smreczak M., Müller T. (2023). Hantavirus Brno loanvirus (*Hantaviridae*) is highly specific to the common noctule bat (*Nyctalus noctula*) and widespread in Central Europe. Virus Genes.

[38] Salmón-Mulanovich G., Vásquez A., Albújar C., Guevara C., Laguna-Torres V.A., Salazar M., Zamalloa H., Cáceres M., Gómez-Benavides J., Pacheco V. (2009). Human rabies and rabies in vampire and nonvampire bat species, southeastern Peru, 2007. Emerg. Infect. Dis..

[39] Baker K.S., Tachedjian M., Barr J., Marsh G.A., Todd S., Crameri G., Crameri S., Smith I., Holmes C.E.G., Suu-Ire R. (2020). Achimota Pararubulavirus 3: A New Bat-Derived Paramyxovirus of the Genus Pararubulavirus. Viruses.

[40] Williamson M.M., Hooper P.T., Selleck P.W., Westbury H.A., Slocombe R.F. (2020). Experimental hendra virus infectionin pregnant guinea-pigs and fruit bats (*Pteropus poliocephalus*). J. Comp. Pathol..

[41] Middleton D.J., Morrissy C.J., van der Heide B.M., Russell G.M., Braun M.A., Westbury H.A., Halpin K., Daniels P.W. (2007). Experimental Nipah virus infection in pteropid bats (*Pteropus poliocephalus*). J. Comp. Pathol..

