# Supplementary material for: Paramyxoviruses in Bats in Poland—The First Detection
Source: Pathogens. 2026 Feb 17;15(2):223. doi: 10.3390/pathogens15020223 (PMC12942713; doi:10.3390/pathogens15020223)
Supplement: Supplementary file 1 [file pathogens-15-00223-s001.zip › supplementary figures_legend.pdf]

**Figure S1.** Phylogenetic tree of bat paramyxoviruses (Bat-PV's) based on 369 bp fragment from RNA polymerase (L) gene. The Polish bat paramyxovirus sequences obtained in the current study are labelled BtParV_sample_ID_POL (red square). Accession numbers for sequences from GenBank are listed for each sequence. The evolutionary history was inferred by using the Maximum Likelihood method and General Time Reversible model. The tree with the highest log likelihood (-29254.89) is shown. The percentage of trees in which the associated taxa clustered together is shown next to the branches. Initial tree(s) for the heuristic search were obtained automatically by applying Neighbor-Join and BioNJ algorithms to a matrix of pairwise distances estimated using the Maximum Composite Likelihood (MCL) approach, and then selecting the topology with superior log likelihood value. A discrete Gamma distribution was used to model evolutionary rate differences among sites (4 categories (+*G*, parameter = 1.7281)). The rate variation model allowed for some sites to be evolutionarily invariable ([+*I*], 0.55% sites). The tree is drawn to scale, with branch lengths measured in the number of substitutions per site. This analysis involved 376 nucleotide sequences. All positions with less than 95% site coverage were eliminated, i.e., fewer than 5% alignment gaps, missing data, and ambiguous bases were allowed at any position (partial deletion option). There were a total of 361 positions in the final dataset. Evolutionary analyses were conducted in MEGA X. Bootstrap over 70% indicated in the tree branches supports the reliability of the tree topology.

**Figure S2. Identity matrix for bat paramyxoviruses (Bat-PV's) used in the study.** Heatmap showing percent identity between pairs of Bat-PV partial RNA polymerase (L) sequences. Sequences are aligned along the x and y axes. Identity scores for each pairwise comparison are represented by coloured boxes with identity increasing from blue to red.

**Figure S3.** Phylogenetic tree of all European bat paramyxoviruses (Bat-PV's) based on 369 bp fragment from RNA polymerase (L) gene. The Polish bat paramyxovirus sequences obtained in the current study are labelled BtParV_sample_ID_POL (red square)_year_of_isolation_host. Accession numbers for sequences from GenBank are listed for each sequence. The evolutionary history was inferred by using the Maximum Likelihood method and Tamura 3-parameter model. The tree with the highest log likelihood (-4594.77) is shown. The percentage of trees in which the associated taxa clustered together is shown next to the branches. Initial tree(s) for the heuristic search were obtained automatically by applying Neighbor-Join and BioNJ algorithms to a matrix of pairwise distances estimated using the Tamura 3 parameter model, and then selecting the topology with superior log likelihood value. A discrete Gamma distribution was used to model evolutionary rate differences among sites (5 categories (+*G*, parameter =

3.0611)). The tree is drawn to scale, with branch lengths measured in the number of substitutions per site. This analysis involved 31 nucleotide sequences. All positions with less than 90% site coverage were eliminated, i.e., fewer than 10% alignment gaps, missing data, and ambiguous bases were allowed at any position (partial deletion option). There were a total of 357 positions in the final dataset. Evolutionary analyses were conducted in MEGA X. Bootstrap over 70% are indicated in the tree branches supporting the reliability of the tree topology.

**Figure S4.** International haplotype network analysis based on 370 sequences of partial (402 bp) RNA polymerase (L) of bat paramyxoviruses (Bat-PV's). The sequences included four sequences from the current study and 366 international Bat-PV sequences obtained from GenBank database. The number of nucleotide substitutions between haplotypes is represented by ticks on branches. Nodes are scaled based on the number of representative sequences and coloured by geographic region of origin. The AMOVA results corresponding to this network are shown in Table 2.
