# Supplementary figures and images for: Paramyxoviruses in Bats in Poland—The First Detection
Source: Pathogens. 2026 Feb 17;15(2):223. doi: 10.3390/pathogens15020223 (PMC12942713; doi:10.3390/pathogens15020223)

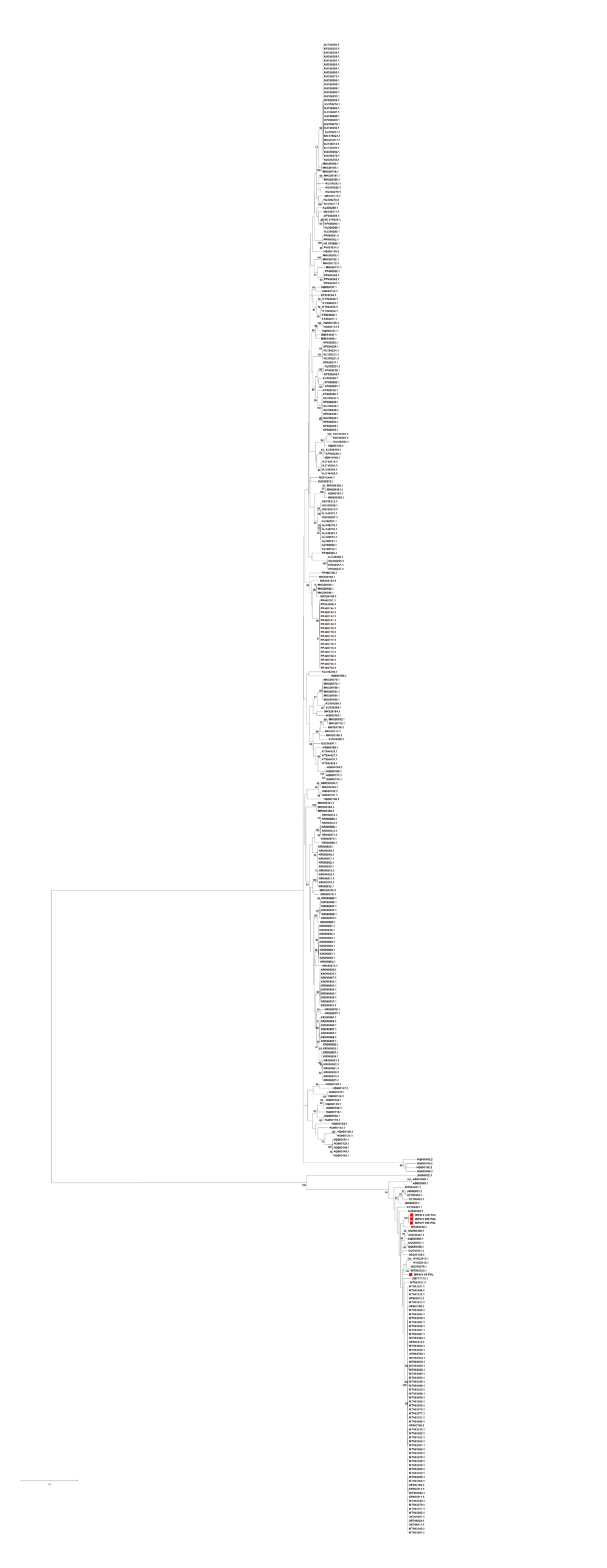

Supplement: Supplementary file 1 [file pathogens-15-00223-s001.zip › Figure S1_all_additional_figure.png]

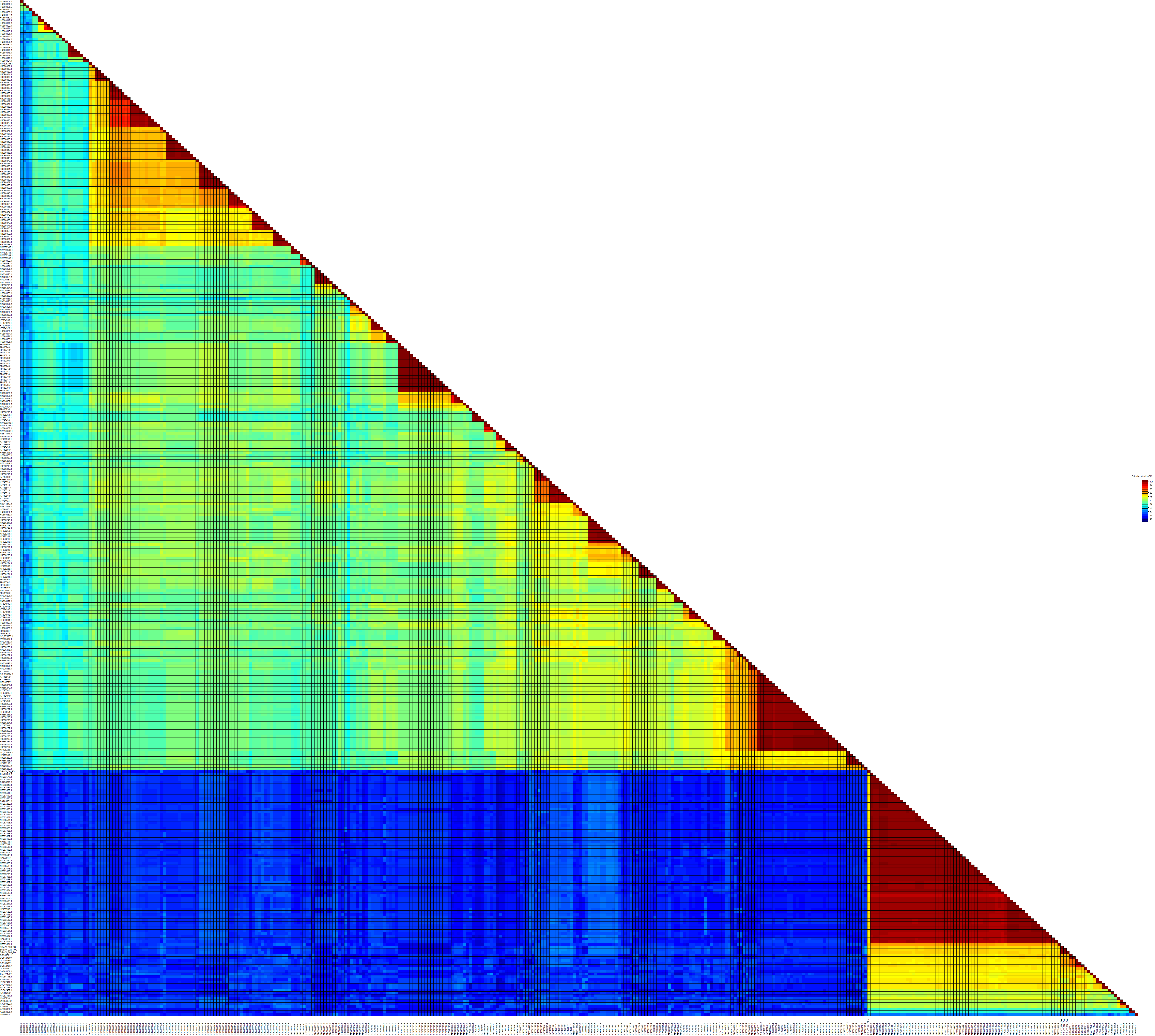

Supplement: Supplementary file 1 [file pathogens-15-00223-s001.zip › Figure S2_Heatmap_additional_figure.png]

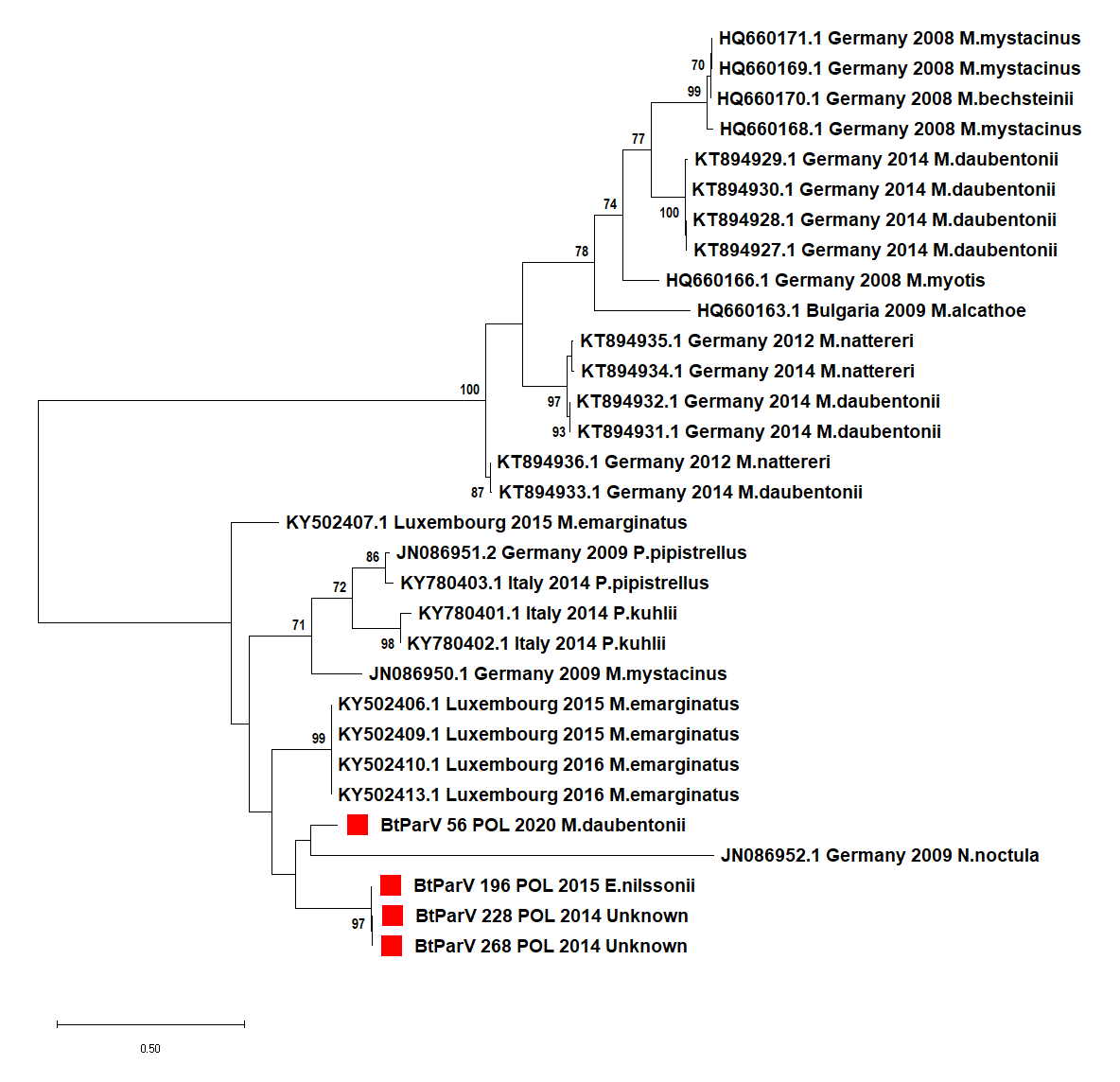

Supplement: Supplementary file 1 [file pathogens-15-00223-s001.zip › Figure S3_Europejskie_15_12_2025.png]

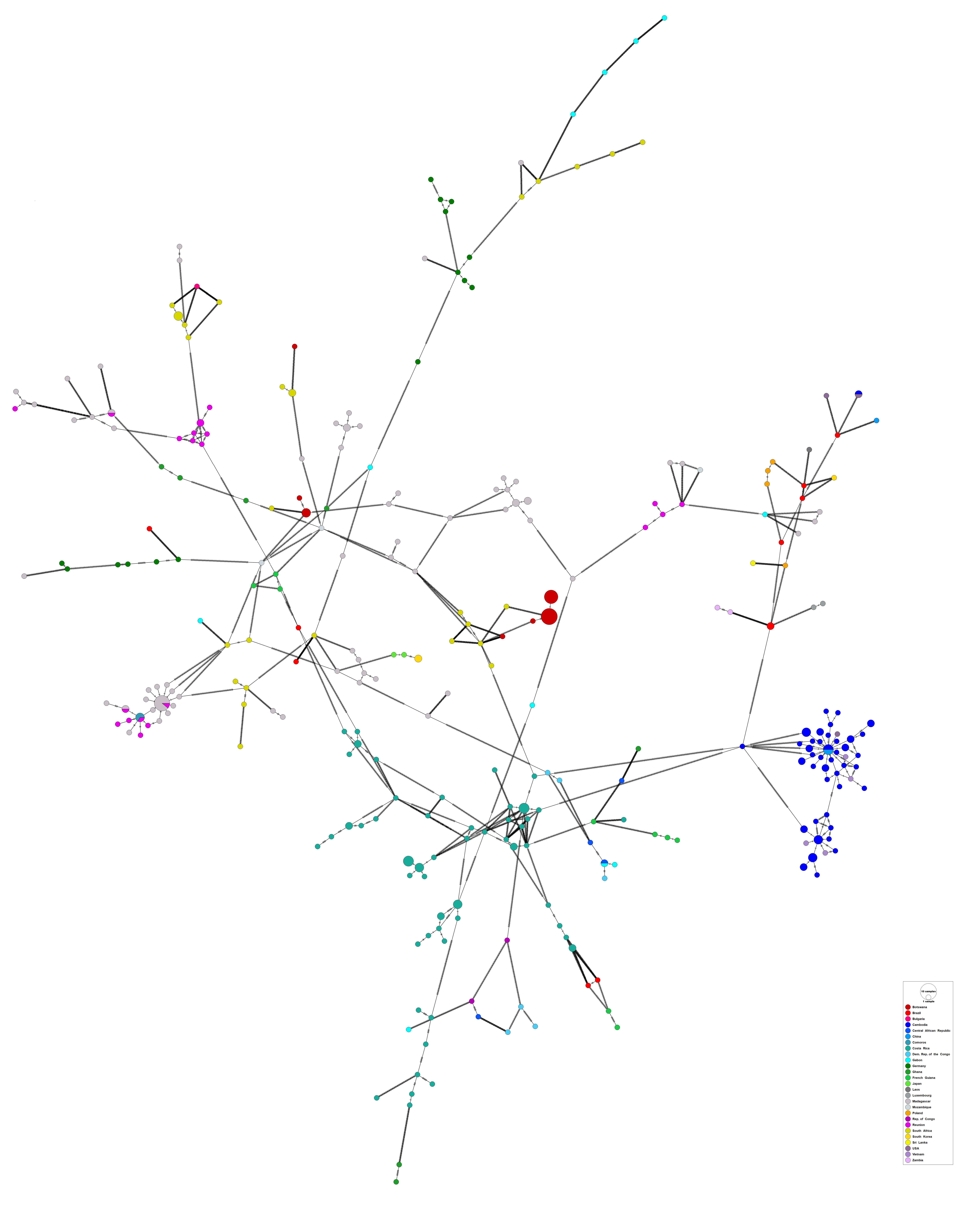

Supplement: Supplementary file 1 [file pathogens-15-00223-s001.zip › Figure S4_Network_additional_figure.png]
